# RNA-Loaded Nanoparticles for Targeted Lung Delivery

**DOI:** 10.3390/biomedicines14051069

**Published:** 2026-05-08

**Authors:** Mark John Siringan, Xiaoyang Chen, Jiawei Huo

**Affiliations:** 1 Feinberg School of Medicine, Northwestern University, Chicago, IL 60611, USA; 2 Malnati Brain Tumor Institute, Robert H. Lurie Comprehensive Cancer Center, Feinberg School of Medicine, Northwestern University, Chicago, IL 60611, USA

**Keywords:** RNA therapeutics, nanoparticle delivery, lung targeting, lung cancer

## Abstract

The lung represents a promising yet underexploited target for RNA therapeutics due to its large surface area and accessibility via non-invasive inhalation delivery. Despite rapid advances in RNA-based modalities, including small interfering RNA (siRNA), microRNA (miRNA), messenger RNA (mRNA), and CRISPR-Cas systems, efficient pulmonary delivery remains a major challenge. Multiple biological barriers, such as mucus and surfactant layers, mucociliary clearance, immune surveillance, and limited cellular uptake of negatively charged nucleic acids, significantly restrict therapeutic efficacy. In addition, aerosolization processes may introduce mechanical stress, compromising RNA integrity. Nanoparticle-based delivery systems have emerged as a central strategy to address these limitations. By protecting RNA cargo, enhancing mucus penetration, and promoting cellular internalization, engineered nanoparticles enable more effective pulmonary delivery. In this review, we adopt a barrier-centered perspective to examine the key biological obstacles to lung-targeted RNA delivery and highlight recent advances in nanoparticle-mediated strategies, with a focus on lipid nanoparticles, polymeric systems, and inorganic nanomaterials. We further discuss design principles that govern RNA stability, transport, and intracellular release and critically compare the strengths, limitations, and translational potential of each platform, including considerations of toxicity, biodegradability, and clinical readiness. Finally, we outline emerging clinical applications of RNA-loaded nanoparticles, using lung cancer as a representative disease model, and discuss remaining challenges and future directions. Continued innovation in nanoparticle engineering and delivery strategies is expected to accelerate the clinical translation of RNA therapeutics for pulmonary diseases.

## 1. Introduction

Respiratory diseases represent a major global health burden [1]. Since 1990, the global number of individuals living with chronic respiratory diseases has increased by nearly 40%, highlighting the growing need for improved therapeutic strategies [2]. In addition, lung cancer has remained the leading cause of cancer-related mortality in both men and women for several decades [3]. Together, these conditions underscore the significant clinical impact of pulmonary diseases and the urgent need for new treatment approaches. For therapeutic development, the lungs represent an attractive target organ due to their large surface area, high vascularization, and potential for localized delivery through inhalation [4]. These features enable high local bioavailability while reducing systemic exposure, making pulmonary delivery an appealing strategy for treating respiratory diseases.

RNA-based therapeutics have emerged as promising tools for precision medicine because they enable highly specific modulation of gene expression [5]. By targeting the underlying genetic drivers of disease, RNA therapies offer the potential for potent and long-lasting therapeutic effects. These approaches can suppress disease-promoting genes or restore protective pathways, including those involved in immune regulation [6]. Several classes of RNA therapeutics have been developed, including small interfering RNA (siRNA), microRNA (miRNA), messenger RNA (mRNA), and clustered regularly interspaced short palindromic repeats (CRISPR)-based gene-editing technologies [7]. Each of these platforms utilizes RNA molecules to achieve sequence-specific targeting of genes or regulatory pathways.

Despite their therapeutic potential, RNA-based treatments face significant challenges when applied to pulmonary delivery. Naked RNA molecules are inherently unstable and susceptible to enzymatic degradation and mechanical fragmentation [8]. Furthermore, the lung contains multiple physiological barriers that limit the effectiveness of nucleic acid therapies [9,10]. These include mucus layers that restrict particle diffusion, mucociliary clearance mechanisms that remove foreign materials, and immune defenses that recognize and eliminate nucleic acids. Surfactant-associated nucleases can also degrade RNA molecules within the airway environment. In addition, the negatively charged phosphate backbone of RNA creates electrostatic repulsion at the cell membrane, further limiting uptake into epithelial cells. Together, these barriers present substantial challenges for the effective application of RNA therapeutics in pulmonary diseases.

One strategy to overcome these limitations is the use of carrier systems that protect and guide RNA molecules to their intended targets. Nanoparticles have emerged as promising delivery vehicles capable of encapsulating or complexing RNA therapeutics while improving their stability and transport [11,12]. Biomaterial-based nanoparticles, particularly lipid nanoparticles, have gained considerable attention due to their biocompatibility and ability to facilitate membrane fusion and cellular uptake [13]. Polymeric nanoparticles represent another class of delivery systems that offer greater synthetic flexibility, allowing for extensive surface modification and rational design for targeted delivery and controlled release [14,15]. Inorganic nanoparticles are also being explored due to their structural rigidity and stability, which can enhance the protection of RNA payloads [16]. Some inorganic materials additionally possess unique optoelectronic properties that can be leveraged for combination therapies, such as photothermal or photodynamic treatment. Together, these nanoparticle platforms provide versatile strategies for stabilizing RNA therapeutics and improving their delivery to lung tissues.

There have been recent excellent reviews that provide comprehensive overviews of lung-targeted RNA delivery systems and their clinical translation [17]. In contrast to prior reviews that primarily summarize nanoparticle platforms and their biological performance, we focus on how specific physicochemical and molecular design features of these systems influence their ability to overcome defined pulmonary delivery barriers. We also expand the discussion to include inorganic nanoparticle platforms that are less frequently addressed and discuss individual RNA modalities as distinct therapeutic categories, rather than focusing primarily on the delivery vehicles. We first examine the limitations associated with inhalable RNA formulations, including aerosolization-induced fragmentation. We then review the extracellular and cellular barriers that restrict the effective delivery of naked RNA within the pulmonary environment. Next, we summarize recent developments in nanoparticle platforms, such as lipid nanoparticles, polymeric nanoparticles, and inorganic nanomaterials, which have been engineered to improve RNA stability, transport, and cellular delivery. Finally, we discuss emerging RNA-loaded nanoparticle therapies for lung cancer as a clinically relevant example and consider the broader implications of these technologies for lung-targeted precision medicine.

In the studies discussed throughout this review, nanoparticle delivery to the lung is achieved through multiple administration routes, including inhalation-based methods such as nebulization or intratracheal instillation, as well as systemic approaches such as intravenous injection. Inhalation and intratracheal delivery enable direct deposition within the airways and alveoli, while intravenous administration relies on nanoparticle design features to promote preferential accumulation within lung tissue following circulation.

## 2. Biological Barriers to RNA Delivery in the Lung

### 2.1. Aerosolization Induces RNA Fragmentation

Lung cancer, along with other pulmonary diseases, presents a unique therapeutic opportunity in which drugs can be administered directly to the respiratory tract rather than through systemic injection [4]. Inhalation-based delivery allows therapeutics to be deposited directly within the airways and alveoli, providing access to an extensive respiratory surface area that can support high local bioavailability and less first-pass metabolism compared with other non-invasive routes of administration [18,19]. Delivering drugs directly to the site of pulmonary pathology may also enable faster therapeutic onset while limiting systemic exposure.

To generate inhalable therapies, liquid formulations must first be converted into aerosols using aerosolization technologies. Common devices include vibrating mesh nebulizers and jet nebulizers, both of which produce inhalable droplets through mechanical processes [20]. While these devices are widely used in pulmonary drug delivery, the forces involved in aerosol generation can pose challenges for nucleic acid therapeutics. In particular, the atomization process exposes molecules to shear stress and repeated air–liquid interface interactions, both of which can compromise the integrity of nucleic acid backbones prior to administration [21,22]. Repeated exposure to the air–liquid interface can promote molecular unfolding and destabilization, as interfacial adsorption disrupts secondary structure and increases susceptibility to fragmentation. Several studies have demonstrated the sensitivity of nucleic acids to shear-induced degradation during nebulization. Catanese Jr. et al. showed that supercoiled DNA plasmids are highly susceptible to fragmentation during jet nebulization, with degradation strongly dependent on plasmid size [23]. Plasmids measuring 5302 base pairs exhibited a half-life of approximately 1.5 min under nebulization conditions, whereas smaller plasmids of 1243 base pairs or less displayed half-lives exceeding 30 min. Many therapeutic mRNA constructs fall within a similar or even larger size range. For instance, the mRNA sequences used in the BNT162b2 and mRNA-1273 SARS-CoV-2 vaccines are approximately 4000 nucleotides in length [24]. mRNA molecules are single-stranded, and their large size and comparatively lower structural stability suggest that they may be particularly vulnerable to mechanical fragmentation during aerosolization.

Consistent with this expectation, van Rijn et al. reported that common nebulization approaches, including vibrating mesh and jet nebulizers, can induce substantial fragmentation of the mRNA contained within BNT162b2 and mRNA-1273 formulations [25]. Similarly, Kim et al. observed significant degradation of firefly luciferase (Fluc) mRNA (~1900 nucleotides) following vibrating mesh nebulization [26]. Together, these findings suggest that the aerosolization step itself may represent an early barrier for inhalable RNA therapeutics, as degradation can occur even before the molecules encounter biological defenses within the lung. Overall, these challenges highlight the need for protective delivery vehicles that can promote RNA stability during aerosolization.

### 2.2. Mucociliary Clearance and Lung Secretions Facilitate Lung Clearance

The lungs possess several innate defense mechanisms designed to capture and remove foreign materials, including therapeutic nucleic acids. A key component of this defense system is the airway mucus layer, a complex viscoelastic fluid that coats much of the respiratory tract. Mucus is composed primarily of water, inorganic salts, lipids, and heavily glycosylated proteins known as mucins, particularly MUC5AC and MUC5B [27,28]. These components create a hydrated biopolymer network that acts as an important physical barrier against inhaled particles and pathogens. Mucus also plays a central role in the mucociliary clearance system. Coordinated beating of ciliated epithelial cells transports mucus and any entrapped material toward the oropharynx, where it can be swallowed or expelled through coughing [29]. While this mechanism is essential for pulmonary host defense, it may also limit the retention of inhaled therapeutics. The physical properties of mucus can be further altered in disease states. Namely, conditions such as cystic fibrosis are associated with elevated levels of extracellular DNA and F-actin filaments within airway secretions, which significantly increase mucus viscoelasticity, enhancing its ability to trap inhaled materials [27].

**Figure 1:**
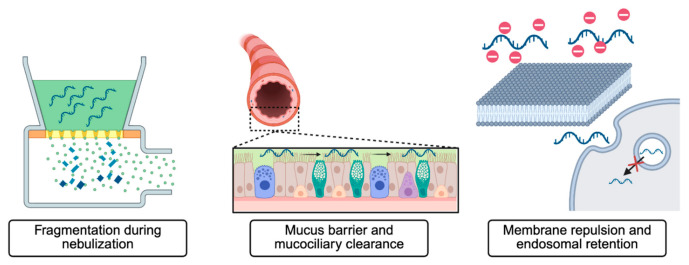
Schematic illustration of barriers to RNA delivery in the lung, including fragmentation during nebulization, mucus barrier and mucociliary clearance, and membrane repulsion with endosomal retention. Created in BioRender. Siringan, M. J. (2026). https://BioRender.com/swdaxuc (accessed on 9 April 2026).

These structural properties of mucus present several challenges for RNA therapeutics (Figure 1). Larger or structurally complex RNA constructs may become physically entangled within the mucin polymer network, slowing their diffusion through the airway mucus layer. In addition to this steric hindrance, electrostatic interactions may also influence RNA transport. Mucins are capped at their termini with negatively charged sialic acid residues, giving the mucus matrix a net negative charge [30]. As a result, electrostatic repulsion between mucins and the negatively charged phosphate backbone of RNA molecules may further hinder the movement of naked RNA therapeutics toward underlying epithelial surfaces. Nanoparticle transport through mucus is also strongly dependent on particle size and surface chemistry, as diffusion through the mucin network is governed by steric hindrance and hydrodynamic interactions. Particles larger than the characteristic pore size of the mucus mesh or those with adhesive surface properties exhibit significantly reduced mobility [31]. Beyond the conducting airways, the distal alveoli are lined by a thin layer of pulmonary surfactant. This surface-active film is composed predominantly of phospholipids, particularly phosphatidylcholines, along with smaller amounts of proteins and other macromolecules [32]. Although surfactant plays an essential role in reducing alveolar surface tension and maintaining lung stability, it may also influence the transport of therapeutic molecules. Interactions between RNA molecules and surfactant-associated proteins or lipid structures may slow diffusion through the alveolar lining fluid and limit access to underlying cells. Bronchoalveolar lavage fluid (BALF) is a saline wash collected from the lower respiratory tract [33]. Under normal physiological conditions, this fluid contains relatively low levels of nuclease enzymes, suggesting that the extracellular environment of the alveolar lining fluid is not strongly nuclease-rich. However, this environment can change substantially during inflammatory conditions. Pulmonary inflammation, which accompanies many respiratory diseases, has been associated with increased expression and secretion of ribonucleases from alveolar macrophages and other immune cells into the airway and alveolar fluids [34,35]. The resulting increase in nuclease activity may promote degradation of extracellular RNA molecules and further reduce the stability of naked RNA therapeutics in inflamed lung tissue. Mucus entrapment, mucociliary clearance, and inflammation-associated nuclease activity are innate lung defense mechanisms that create a challenging environment for the direct delivery of unprotected nucleic acid therapeutics.

### 2.3. RNA Therapeutics Are Susceptible to Cellular Barriers

In the event that RNA therapeutics are able to penetrate the mucus layer and alveolar surfactant to reach the epithelial lining of the lung, several cellular barriers may still limit their activity. One major obstacle is inefficient cellular uptake. The plasma membrane of epithelial cells carries a net negative charge, which electrostatically repels the negatively charged phosphate backbone of nucleic acids. As a result, naked RNA molecules generally exhibit poor membrane penetration [36].

Experimental studies have highlighted this limitation in pulmonary delivery. Moschos et al. administered siRNA and locked nucleic acids intratracheally in mice and found that, within lung tissue, the nucleic acids were preferentially internalized by alveolar macrophages rather than epithelial cells [37]. This observation reflects the broader challenge for nucleic acid therapeutics. Namely, RNA molecules are large and highly charged macromolecules, and they typically enter cells through endocytic pathways rather than passive diffusion across the membrane [38]. While certain chemically modified oligonucleotides, such as locked nucleic acids, can undergo cellular entry through a process known as gymnosis, this mechanism occurs slowly and generally requires extended incubation times compared with conventional transfection approaches [39,40]. Even when RNA molecules are successfully internalized, their intracellular trafficking poses another barrier. Most nucleic acids that enter cells through endocytosis become trapped within endosomal compartments [41]. To exert their intended biological effect, RNA therapeutics must escape from these endosomes and reach the cytoplasm. However, endosomal escape is notoriously inefficient. For example, it has been reported that as little as 0.01% of GalNAc-modified siRNA molecules internalized by cells ultimately escape from endosomes into the cytoplasm [42]. Molecules that fail to escape are typically trafficked to lysosomes, where the acidic environment and degradative enzymes lead to nucleic acid breakdown.

In addition to these uptake and trafficking barriers, exogenous RNA molecules can also stimulate innate immune responses. Certain RNA sequences are recognized by pattern recognition receptors such as Toll-like receptor 3 (TLR3) and Toll-like receptor 7 (TLR7), which can trigger downstream cytokine production and inflammatory signaling [43,44]. Judge et al. previously demonstrated that siRNA administration can induce an innate cytokine response through such pathways and that certain sequences are inherently more sensitized to this immunostimulation [45]. Similar inflammatory effects have also been implicated in off-target toxicity and reduced gene silencing efficiency in some antisense oligonucleotide and siRNA studies conducted in the lung [37,46]. These findings highlight an inherent challenge of RNA-based therapeutics. While RNA can act as a powerful regulatory molecule, exogenous RNA may still be interpreted by the host immune system as a foreign material.

Taken together, the susceptibility of RNA molecules to degradation during aerosolization, including enzymatic cleavage by ribonucleases and chemical hydrolysis of the phosphodiester backbone, the physiological barriers that limit trafficking through the respiratory tract, and the cellular challenges of inefficient uptake, endosomal trapping, phagocytic clearance, and immune activation, restrict the therapeutic potential of naked RNA in the lung. These limitations have motivated the development of delivery systems designed to stabilize RNA molecules, facilitate cellular uptake, promote endosomal escape, and mitigate immune recognition. Such strategies form the basis of nanoparticle-based RNA delivery platforms.

## 3. Nanoparticle Platforms for RNA Therapeutics

Nanoparticles have emerged as a promising strategy to overcome many of the barriers associated with naked RNA delivery. These nanoscale structures, which can be composed of lipids, polymers, or inorganic materials, are stabilized through various molecular interactions and can be engineered with different sizes, morphologies, and surface properties [47,48]. Nanoparticle size is a key determinant of pulmonary delivery, influencing deposition within the respiratory tract, diffusion through mucus, and the mechanism of cellular uptake, with smaller particles generally favoring deeper lung penetration and endocytic internalization [49]. In the context of RNA therapeutics, nanoparticles can protect nucleic acids from degradation, improve cellular uptake, and help facilitate intracellular delivery [50,51]. These functions are governed by specific physicochemical properties, including surface charge, hydrophobicity, molecular architecture, and functional group composition, which determine how nanoparticles interact with extracellular barriers such as mucus and intracellular processes, including membrane translocation and endosomal escape. As a result, nanoparticle-based systems have become widely studied carriers for RNA therapies. While numerous nanoparticle platforms have been developed, this section focuses on three major classes used for RNA delivery: lipid nanoparticles, synthetic polymer-based nanoparticles, and inorganic nanoparticles.

At the molecular level, ionizable lipids within LNPs play a central role in transfection efficiency by enabling electrostatic complexation with RNA and undergoing pH-dependent protonation within endosomes, which facilitates membrane destabilization and promotes cytosolic release. The pKa of these ionizable lipids is a critical design parameter, as it determines the extent of protonation within endosomal compartments and directly influences the efficiency of endosomal escape [52]. These features allow LNPs to overcome both extracellular degradation in the airway environment and intracellular trafficking barriers following uptake.

### 3.1. Lipid Nanoparticles

**Figure 2:**
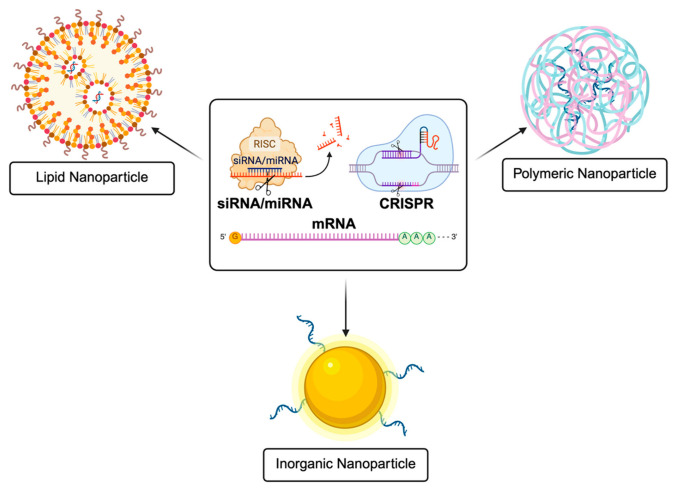
Schematic illustration of nanoparticle platforms for RNA delivery, including lipid nanoparticles, polymeric nanoparticles, and inorganic nanoparticles, and representative RNA therapeutics (siRNA/miRNA, mRNA, and CRISPR systems). Created in BioRender. Siringan, M. J. (2026). https://BioRender.com/uhyapqj (accessed on 9 April 2026).

Lipid nanoparticles (LNPs) are nanoscale structures composed primarily of lipids arranged in a manner that resembles cellular membranes. In typical formulations, ionizable or helper lipids assemble with cholesterol and polyethylene glycol (PEG) lipids to form a particle capable of encapsulating nucleic acids within its interior [53]. These structures offer several advantages for RNA delivery. First, the lipid composition enables efficient interaction with cellular membranes, which can improve the cellular uptake of the encapsulated RNA. Second, the lipid shell provides a physical barrier that can protect nucleic acids from mechanical and chemical degradation during delivery (Figure 2).

**Figure 3:**
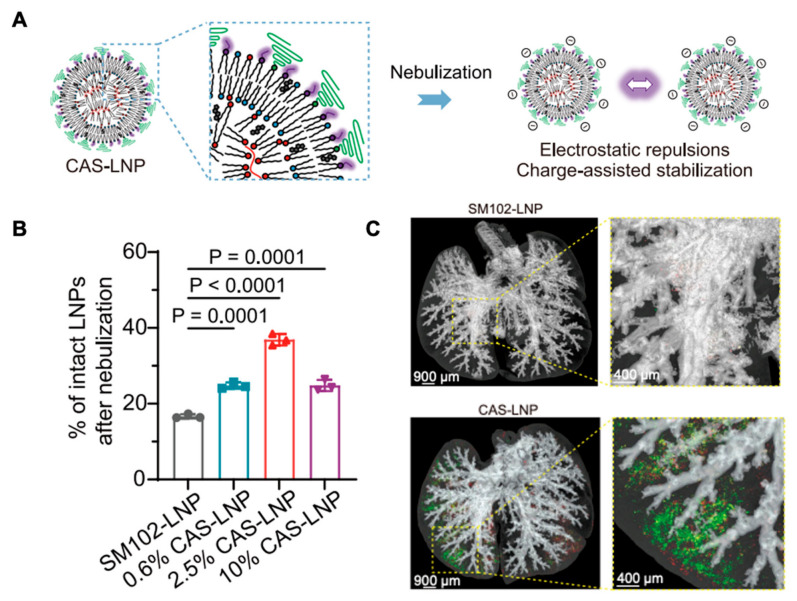
(**A**) Schematic illustrating the preparation and mechanism of CAS-LNP. (**B**) Percentage of intact LNPs after nebulization. (**C**) Three-dimensional fluorescent imaging of lungs in mice treated with inhaled (**top**) SM102-LNP control or (**bottom**) CAS-LNP. Figure adapted from Liu et al. (*Nature Communications*, **2024**, *15*, 9471) [55], licensed under the Creative Commons Attribution—Non-Commercial License (CC BY-NC).

Several studies have demonstrated the ability of LNPs to address the aerosolization challenges described earlier. Zhang et al. showed that LNP formulations could protect encapsulated EGFP mRNA from shear-induced degradation during aerosolization [54]. This protection is mediated by the lipid bilayer-like structure of LNPs, which shields RNA from shear-induced mechanical stress, preserving its structural integrity and maintaining its capacity for downstream translation following cellular uptake. In their study, mice received intratracheal administration of either aerosolized or non-aerosolized LNP-encapsulated mRNA, and the resulting GFP expression in lung tissue was comparable between the two groups. These results suggest that LNPs can effectively shield RNA from fragmentation during the nebulization process. Further work has explored how modifications to lipid composition can improve nanoparticle stability during aerosol delivery. Liu et al. enhanced LNP stability by incorporating a negatively charged DSSC peptide moiety onto a DOPE lipid [55]. This modification increased the zeta potential of the nanoparticles and improved particle dispersion, reducing aggregation that can otherwise promote shear degradation during nebulization (Figure 3A). Following mesh nebulization, these stabilized LNPs showed significantly higher particle recovery compared with non-modified formulations (Figure 3B). The authors also observed that increasing PEG content improved particle transport through mucus, resulting in greater deposition within lung tissue as measured by the fluorescence of Cy5-labeled mRNA (Figure 3C). PEGylation reduces surface charge interactions with mucin glycoproteins, decreasing adhesive trapping and enabling more effective diffusion through the mucus layer, thereby improving access to epithelial cell surfaces where uptake can occur [56].

In addition to inhalation delivery, LNP design can influence organ distribution following systemic administration. Cheng et al. demonstrated that altering the charge of LNPs by varying the proportion of the permanently cationic lipid DOTAP allowed preferential delivery to the lung after intravenous injection [57]. Using a Cre mRNA and CRISPR-Cas9 fluorescent reporter system, the authors found that these lung-targeted LNPs were primarily taken up by epithelial and endothelial cells within the lung, while uptake by immune cells was comparatively limited. Increased surface cationic character enhances electrostatic interactions with negatively charged cellular membranes, promoting uptake, while also altering interactions with proteins in the lung environment, which can reduce recognition and clearance by immune cells [58]. Similar findings were reported by Somu Naidu et al., who developed biodegradable lipids containing different linker chemistries that exposed either positive or negative surface charges [59]. These LNPs were packaged with luciferase-encoding mRNA. Formulations incorporating amide or urea linkers preferentially exposed positive surface charges and showed enhanced lung targeting following retro-orbital administration. These particles also demonstrated greater uptake in epithelial and endothelial cells compared with myeloid or dendritic cells. Consistent with this theme of utilizing positive charges, other studies have also explored sulfonium lipids, which contain a tertiary sulfur that carries a permanent positive charge and have been shown to promote preferential lung sorting and epithelial tissue uptake [60].

Altogether, these studies highlight how LNP platforms can overcome several of the barriers to RNA delivery discussed previously. LNPs can protect RNA cargo during aerosolization, improve stability during nebulization, and enhance transport through mucus. In addition, rational modification of lipid composition can influence tissue distribution and cellular uptake, enabling preferential delivery to lung epithelial and endothelial cells while reducing nonspecific phagocytic clearance. These effects are largely driven by the physicochemical properties of LNP components, including lipid composition, surface charge, and PEG content, which together govern interactions with extracellular barriers such as mucus and intracellular processes such as endosomal escape that ultimately determine transfection efficiency. These properties have established LNPs as one of the most widely explored platforms for pulmonary RNA delivery.

At the molecular level, the clinical success of LNPs can be largely attributed to the intrinsic properties of their lipid components. These particles are composed of biocompatible lipids that readily interact with cellular membranes, enabling efficient uptake through lipid bilayer fusion or endocytosis. This behavior is influenced by lipid packing geometry and hydrophobic interactions, which facilitate membrane fusion and destabilization, allowing RNA cargo to more effectively cross the lipid bilayer barrier. In combination with ionizable lipids that facilitate endosomal escape, this supports high levels of cytosolic delivery and enables more potent therapeutic responses [61]. In the pulmonary setting, these same properties also promote diffusion through airway lining fluids and facilitate uptake by epithelial cells, while pH-dependent membrane destabilization enhances the release of RNA cargo into the cytosol following endocytosis. Efficient cytosolic release is a critical determinant of transfection, as RNA must avoid lysosomal degradation to remain functional for translation or gene editing. These features provide a mechanistic basis for their successful clinical translation, including their widespread use in mRNA vaccines during the COVID-19 pandemic [62].

In practice, LNPs are most appropriate in settings where efficient and scalable RNA delivery is prioritized, particularly for applications requiring high transfection efficiency and established clinical translation pathways. They are well-suited for both systemic delivery strategies that rely on tissue targeting through circulation and inhalation-based approaches, where rapid cellular uptake is desired. However, because LNPs are formed through self-assembly, they lack precise control over surface functionalization [63]. This results in a heterogeneous presentation of targeting ligands, limiting the ability to intentionally cluster ligands or control their spatial distribution. As a result, binding interactions with target cells are often governed by relatively low or variable binding affinity, and opportunities to enhance multivalent avidity are difficult to achieve. This becomes particularly important in the lung, where selective targeting across diverse epithelial and immune cell populations requires finely tuned interactions [64]. Similar to other biological nanoparticles, LNPs exhibit a lower shelf life, requiring very cold storage conditions. They also have a disposition to aggregate, presenting problems for long-term storage.

In addition, LNPs must still encounter physiologic barriers that are not fully resolved by current formulations. Uptake by alveolar macrophages, interactions with pulmonary surfactant, and lipid-dependent inflammatory responses can reduce effective delivery or limit repeat dosing. This process is influenced by nanoparticle size, surface charge, and protein adsorption, all of which affect opsonization and subsequent recognition by phagocytic cells. From a formulation standpoint, maintaining nanoparticle integrity during aerosolization remains a challenge, as shear forces can alter particle structure and compromise RNA stability, even in optimized systems. Together, these chemical and biological constraints highlight the need for improved control over LNP architecture to better match the complexity of pulmonary delivery.

### 3.2. Organic Polymers

Organic polymers represent a broad class of synthetic nanoparticles composed of polymeric units that assemble into defined three-dimensional structures [65]. Similar to lipid nanoparticles, these systems can encapsulate or complex RNA therapeutics and shield them from mechanical and chemical degradation. Their surfaces can also be readily modified to tune physicochemical properties and biological interactions. Compared with biomaterial-based nanostructures such as lipid nanoparticles, polymeric nanoparticles often offer greater synthetic versatility, as individual components can be more easily modified, enabling substantial structural flexibility and functional diversity. From a barrier-centered perspective, this level of synthetic control enables polymeric systems to be designed specifically to overcome extracellular barriers such as mucus and intracellular barriers, including endosomal escape.

Miktoarm star nanoparticles are branching nanostructures composed of polymeric arms such as poly(2-dimethylaminoethyl methacrylate) (PDMAEMA) and poly[oligo(ethylene glycol) methyl ether methacrylate] (POEGMA) [66]. Ma et al. employed Cy5.5-labeled star nanoparticles as carriers for siRNA delivery to the lung [67]. Similar to other nanostructures, these particles were internalized into A549 cells primarily through endocytosis. Notably, their work demonstrated enhanced endosomal escape by optimizing the amount of polymer required to complex siRNA through tuning the nitrogen-to-phosphate (N:P) ratio, where nitrogen comes from the amine group of nanoparticles while phosphate comes from the siRNA backbone. Increasing the nitrogen content improved endosomal escape, suggesting that the polymer-to-siRNA ratio can strongly influence intracellular delivery. This reflects the role of protonatable amine groups in buffering endosomal pH, leading to osmotic swelling and membrane destabilization that facilitates the release of RNA cargo into the cytosol. In mice bearing orthotopically implanted Lewis lung carcinoma (LLC) tumors, the authors also compared systemic and nebulized administration. While systemic delivery resulted in diffuse distribution across organs, including the kidney, liver, spleen, and lung, nebulization produced markedly greater accumulation within lung tissue. Furthermore, nebulized nanoparticles carrying siRNA targeting β3-tubulin and PLK1 achieved measurable gene knockdown in LLC tumors, illustrating the therapeutic potential of inhalable polymeric nanocarriers.

**Figure 4:**
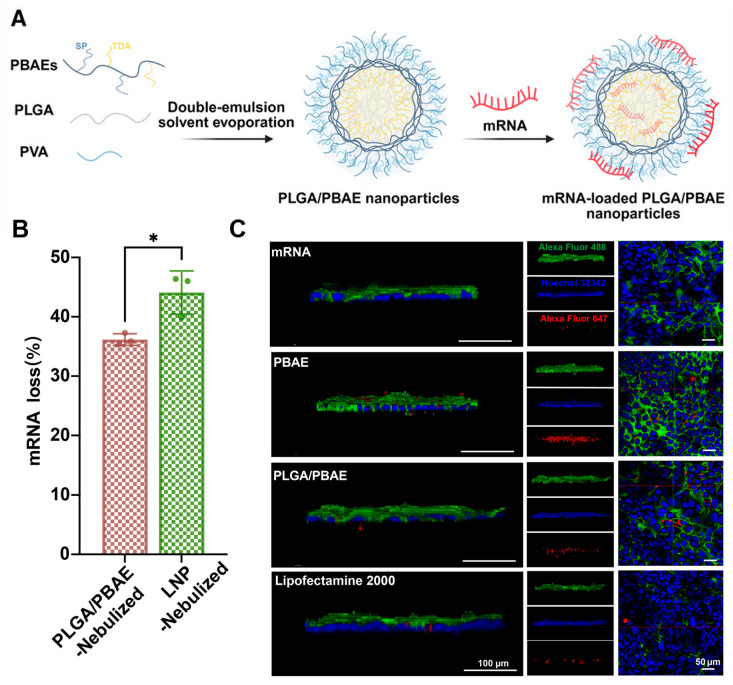
(**A**) Synthesis and structure of hybrid PLGA/PBAE nanoparticle. (**B**) Quantification of mRNA loss during nebulization. Data are presented as mean ± SD, n = 3. *, *p* < 0.05, Student’s *t* test. (**C**) Mucus penetration of different formulations in ALI-cultured Calu-3 cells after 24 h of transfection. AF647-labeled mRNA is shown in red, nuclei in blue, and the mucus layer in green. Scale bar, 100 and 60 µm. Figure adapted from Jiang et al. (*Cell Biomaterials*, **2026**, *2*, 100311) [70], licensed under the Creative Commons Attribution License (CC BY).

Poly(lactic-co-glycolic acid) (PLGA) nanoparticles are biodegradable organic nanostructures that have received FDA approval for various drug delivery applications, including nucleic acid therapeutics [68]. Poly(β-amino esters) (PBAEs) represent another biodegradable polymer family widely used for non-viral nucleic acid transfection [69]. Jiang et al. combined these two platforms to develop a hybrid PLGA/PBAE nanoparticle system for mRNA delivery to the lung (Figure 4A) [70]. Following mesh nebulization, the hybrid particles showed reduced mRNA fragmentation and loss compared with a reference SM102-LNP formulation (Figure 4B). In addition, nebulized PLGA/PBAE nanoparticles produced stronger intracellular EGFP expression than the reference LNP system, suggesting improved preservation of functional mRNA during aerosol delivery. Mechanistically, the hybrid system also enhanced endosomal escape relative to PBAE alone. The authors proposed that PLGA hydrolysis increased PBAE protonation and mRNA condensation, thereby strengthening the proton-sponge effect that facilitates endosomal escape. Using an air–liquid interface Calu-3 epithelial cell model to mimic the airway mucus barrier, the authors further showed that fluorescently labeled hybrid nanoparticles penetrated the mucus layer more effectively than either PBAE or LNP formulations alone (Figure 4C). This demonstrates the ability of polymer design to directly address the airway mucus barrier, which can otherwise limit nanoparticle diffusion and access to epithelial surfaces. Together, these findings suggest that polymeric hybrid systems can improve RNA stability during nebulization while also enhancing mucus penetration and intracellular delivery. Effective intracellular delivery in these systems depends on both efficient uptake and the ability to avoid lysosomal degradation through mechanisms such as proton sponge-mediated endosomal escape.

Metal–organic frameworks (MOFs), which consist of repeating organic ligands coordinated to metal nodes, have also been explored for nucleic acid delivery due to their structural rigidity and stability [71]. Weng et al. demonstrated the use of a zirconium-based porphyrin MOF as a carrier for siRNA delivery [72]. In mice receiving intratracheal administration, the MOF platform produced significantly greater lung accumulation of Cy5-labeled siRNA compared with naked fluorescently labeled siRNA, highlighting the potential of MOF structures to enhance pulmonary delivery. Poly(amine-co-ester) (PACE) polyplexes represent another tunable polymer platform capable of encapsulating and protecting nucleic acids. Suberi et al. developed PEGylated PACE (PACE-PEG) polyplexes for mRNA delivery to the lung [73]. Following intratracheal administration of Fluc mRNA-loaded nanoparticles, both in vivo imaging and explant analysis revealed strong localization within lung tissue. The nanoparticles were predominantly observed within the large airway and alveolar regions and were particularly associated with epithelial cells. Additional studies have demonstrated how rational polymer design can further enhance lung targeting. Tiwade et al. modified polyethylenimine (PEI), a widely used cationic gene delivery polymer, by reacting it with hydrophobic tails to form amino acrylate nanostructures capable of self-assembly [74]. These particles retained high amine density for RNA complexation while incorporating PEG groups to improve stability. Compared with naked RNA and a reference LNP formulation, the resulting nanoparticles delivered Fluc mRNA more efficiently to the lung following intravenous administration.

Similarly, Liu et al. developed lung-specific supramolecular nanoparticles (LSNPs) through the assembly of a platinum-based metal–organic polyhedron capped with hydrophobic adamantane groups and β-cyclodextrin-functionalized PEI [75]. Hydrophobic interactions between adamantane and the polyhedral core drove nanoparticle assembly, while PEG chains improved stability. When loaded with a CRISPR-Cas13d system targeting Cathepsin L and administered intravenously, the LSNP platform produced selective gene knockdown in the lung while leaving expression levels in other organs largely unchanged.

These studies highlight organic polymer nanoparticles as a versatile alternative to lipid nanoparticles for pulmonary RNA delivery. Their modular chemistry allows extensive control over structure, charge, and functional groups, enabling rational design strategies that enhance RNA complexation, protect nucleic acids from degradation, and promote mucus penetration and endosomal escape. Through both inhalation and systemic administration routes, these materials have shown the capacity to improve lung targeting and facilitate RNA delivery to epithelial tissues, underscoring their potential in the development of RNA therapeutics for lung disease.

Because these materials are built through defined chemical routes rather than self-assembly, their backbone composition, charge distribution, and functional groups can be tuned with a high degree of precision [76]. In addition, the inherent flexibility of polymer chains allows for dynamic conformational flexibility, which can facilitate interactions with biological barriers such as mucus networks and cellular membranes. This allows more deliberate control over RNA complexation and enables targeting strategies in which ligand density and spatial presentation can be adjusted to influence binding affinity and enhance multivalent avidity through ligand clustering. The presence of tunable cationic groups also facilitates electrostatic interaction with negatively charged nucleic acids, promoting stable complex formation and cellular uptake, while buffering capacity within certain polymer systems can enhance endosomal escape through proton sponge-mediated mechanisms [77]. These structures are also amenable to direct characterization techniques such as nuclear magnetic resonance spectroscopy (NMR), enabling more precise correlation between molecular structure and delivery performance.

Polymeric nanoparticles may be preferred in scenarios where greater control over nanoparticle structure and surface functionality is required, particularly when targeting specific cell populations or overcoming complex delivery barriers such as dense mucus or intracellular trafficking limitations. Their tunability makes them well-suited for applications that require more deliberate, barrier-driven design rather than broadly efficient delivery. From a formulation standpoint, polymer composition can be adjusted to address key physiological barriers in the lung. Increasing backbone hydrophilicity can reduce aggregation and improve colloidal stability, while PEG incorporation and other modifications can enhance mucus penetration and limit nonspecific interactions. Compared with lipid-based systems, many synthetic polymer nanoparticles also exhibit greater shelf stability, which may be advantageous for long-term storage and handling. These features, taken together, make polymer platforms particularly well-suited for rational, barrier-informed design.

At the same time, these benefits come with practical tradeoffs. The synthesis and functionalization of complex polymer structures often require multistep reactions that can be time- and resource-intensive, sometimes resulting in lower overall yields and higher production costs. In addition, achieving the right balance between delivery efficiency and biocompatibility remains a challenge, particularly for cationic systems that can introduce cytotoxicity or inflammatory responses. These considerations continue to limit widespread clinical adoption, even as polymer-based systems offer clear advantages from a design perspective.

### 3.3. Inorganic Nanoparticles

Inorganic nanoparticles represent a class of metal-based nanomaterials characterized by high structural stability and tunable physicochemical properties [78]. These systems can be engineered with a wide range of morphologies, sizes, and surface chemistries, allowing for flexible design in drug delivery applications. From a barrier-centered perspective, the performance of inorganic nanoparticles is largely determined by how their material properties influence interactions with extracellular barriers such as mucus and intracellular processes, including cellular uptake and endosomal escape. Among the many inorganic nanomaterials explored for nucleic acid delivery, gold nanoparticles and mesoporous silica nanoparticles are two of the most studied platforms.

Gold nanoparticles consist of clusters of gold atoms that can assemble into a variety of geometries, including spheres, rods, cages, stars, and prisms [79]. Their surfaces can be readily functionalized with ligands, polymers, and nucleic acids, making them attractive carriers for gene delivery [80]. Conde et al. demonstrated the use of gold nanoparticles functionalized with RGD peptides for tumor targeting, PEG groups for stability, and siRNA for silencing c-Myc [81]. In cell culture experiments using the lung adenocarcinoma cell line LA-4, treatment with siRNA-loaded gold nanoparticles resulted in significantly greater suppression of c-Myc expression compared with naked siRNA. These findings suggest that the nanoparticle formulation improved cellular delivery of the siRNA cargo, helping to overcome the barrier of poor membrane penetration. This enhancement is primarily driven by surface functionalization and charge-mediated interactions with cellular membranes, which promote endocytic uptake and increase intracellular delivery of the siRNA cargo. In vivo studies further supported these results. Following intratracheal administration of the siRNA-loaded nanoparticles in mice bearing LA-4 tumors, the authors observed a significant reduction in tumor size by immunohistochemical analysis, indicating that the gold nanoparticle platform was capable of protecting the siRNA within the lung environment and enabling delivery to tumor tissue.

Liu et al. reported similar findings using gold nanoprisms functionalized with siRNA targeting PD-L1 [82]. In lung adenocarcinoma HCC827 cells, the siRNA-loaded nanoprisms demonstrated significantly greater cellular uptake compared with free siRNA. Increased uptake in these systems is similarly attributed to surface interactions that facilitate endocytosis, helping to overcome cellular entry as a key barrier to delivery. Moreover, after intravenous administration in mice bearing lung tumor xenografts, the nanoparticles exhibited preferential accumulation in lung tissue. These findings further highlight the ability of gold nanoparticle platforms to facilitate cellular uptake and enhance the delivery of nucleic acid therapeutics across pulmonary barriers. Other studies have explored additional gold nanostructures, including nanocages, which can be functionalized with stabilizing polymers and targeting ligands to further improve nucleic acid delivery [83].

Mesoporous silica nanoparticles represent another class of inorganic delivery systems that have attracted interest for RNA therapeutics [84]. These particles are composed of amorphous silica and are characterized by a highly porous structure that provides a large surface area and high drug-loading capacity. Chen et al. developed mesoporous silica nanoparticles encapsulating siRNA within their pores and coated the particle surface with PEG to improve stability [85]. When administered to A549 tumor-bearing mice via intravenous injection, the silica nanoparticle system showed greater accumulation of siRNA in lung tissue compared with naked siRNA, suggesting that the nanoparticle formulation improved delivery across pulmonary barriers. The porous structure of these nanoparticles enables high RNA loading and gradual release, while surface coatings such as PEG reduce aggregation and improve transport through airway lining fluids, facilitating access to target cells [86]. Similar observations were reported by Dilnawaz et al., who demonstrated significantly higher uptake of siRNA-loaded mesoporous silica nanoparticles in A549 cells compared with free siRNA [87].

Overall, these studies indicate that inorganic nanoparticles can serve as effective carriers for nucleic acid therapeutics in the lung. Although they have been explored less extensively than lipid or polymeric systems for pulmonary gene delivery, inorganic nanoparticles offer several advantages, including strong structural stability, tunable surface functionalization, and the ability to enhance cellular uptake and protect RNA cargo. These effects are influenced by surface chemistry and particle geometry, which modulate interactions with cell membranes and can promote internalization through energy-dependent endocytic pathways. Through these properties, inorganic nanoparticles can overcome key extracellular barriers, including degradation in the lung environment and limited cellular access, making them promising alternative platforms.

Beyond these general advantages, inorganic nanoparticle platforms are distinguished by their underlying material properties. Their rigid and highly ordered structures allow for consistent control over size and morphology, while surface functionalization enables the attachment of targeting ligands, polymers, or nucleic acids. In addition, many inorganic systems possess optoelectronic properties that can be leveraged for combined therapeutic and diagnostic applications, such as photothermal or photodynamic therapy, which may be particularly useful in oncologic settings [88,89]. Their structural stability also contributes to the protection of RNA cargo and supports cellular uptake across pulmonary barriers. Surface charge and functional coatings can further facilitate interactions with cellular membranes, promoting endocytic uptake, while in certain systems, externally triggered or surface-mediated effects may enhance endosomal disruption and intracellular release of RNA cargo. These mechanisms are critical for overcoming intracellular trafficking barriers that limit effective RNA transfection.

Inorganic nanoparticles are most appropriately applied in specialized contexts, particularly in oncologic applications where their structural stability and optoelectronic properties can be leveraged for combined therapeutic and diagnostic strategies. They may be advantageous when multimodal functionality is desired, although their use is more limited in settings requiring repeated dosing or long-term biocompatibility. However, these same features also impose important constraints. Similar to LNPs, inorganic nanoparticles are often functionalized through surface chemistry that does not allow precise control over ligand attachment, valency, or spatial organization. As a result, ligand presentation is inherently heterogeneous, making it difficult to systematically tune binding affinity or enhance multivalent avidity and introducing potential variability in biological interactions and therapeutic response. Compared with polymeric systems, these platforms therefore offer less control at the level of rational design.

More importantly, many inorganic materials are not readily biodegradable, raising concerns about long-term accumulation and toxicity in lung tissue. This has been observed in certain systems, including gold nanoparticles, where persistence within tissues has been associated with inflammatory responses in preclinical models [90,91]. Formulation can also present barriers, as some inorganic systems exhibit low solubility, which can promote aggregation and reduce colloidal stability over time, ultimately limiting shelf life and reproducibility. This behavior is often driven by high surface energy and unfavorable solvent interactions, which promote particle–particle association and limit dispersion stability in biological environments. In addition, inorganic nanoparticles must still navigate physiologic barriers such as mucus, surfactant interactions, and phagocytic clearance without the same degree of tunability available in polymer-based systems. Together, these limitations have slowed clinical translation despite promising preclinical results and suggest that inorganic nanoparticles may be best suited for specialized or multimodal applications rather than as broadly applicable RNA delivery platforms. These features indicate that inorganic nanoparticles can address multiple stages of RNA delivery, including extracellular stability, cellular uptake, and intracellular release, although with less tunability compared to polymeric systems.

Taken together, these platforms reflect different tradeoffs between chemical control and clinical readiness. These differences ultimately arise from how each platform is constructed at the molecular level, with self-assembled lipid systems favoring translational efficiency, chemically defined polymers enabling precise structure-function tuning, and inorganic materials offering stable but less adaptable architectures. Lipid nanoparticles remain the most established option, largely due to their demonstrated performance at scale, most clearly during the COVID-19 pandemic. Their biocompatible lipid composition, efficient interaction with cellular membranes, and consistent endosomal escape have enabled reliable RNA delivery in vivo. At the same time, their reliance on self-assembly limits control over how ligands are presented on the particle surface, making it difficult to systematically tune binding affinity or take advantage of multivalent avidity for more selective targeting. Polymeric nanoparticles offer a different approach, with greater control over structure and functionalization that allows for more deliberate, barrier-focused design, particularly in the complex lung environment. Inorganic systems are less advanced clinically but remain relevant in more specialized contexts, especially where their physical properties, such as optical responsiveness or structural stability, can be used alongside RNA delivery. Overall, lipid nanoparticles currently represent the most practical platform for clinical application, while polymer-based systems may offer additional opportunities to refine targeting and delivery as design strategies continue to evolve.

## 4. RNA Therapies in Lung Cancer

A variety of RNA therapies have been explored for the treatment of pulmonary diseases. Among these, many studies have focused on lung cancer, which remains the leading cause of cancer-related mortality worldwide in both men and women [92]. Many genetic drivers and regulatory pathways involved in lung cancer development have been identified, making lung cancer an attractive target for gene-based therapies [93,94]. In this section, we highlight the major classes of RNA therapeutics and recent advances in nanoparticle-mediated delivery strategies developed to target lung cancer.

### 4.1. Small-Interfering RNA

Small interfering RNA (siRNA) is a short double-stranded RNA molecule, typically 20–25 base pairs in length, consisting of a guide strand and a passenger strand [95]. The guide strand is complementary to a target messenger RNA (mRNA) sequence and directs the RNA-induced silencing complex (RISC) to the transcript. Upon binding, the target mRNA is cleaved and subsequently degraded, resulting in the suppression of gene expression. Because many oncogenic pathways in lung cancer are driven by overexpressed or dysregulated genes, siRNA therapeutics offer a strategy to selectively silence these targets and inhibit tumor progression.

**Figure 5:**
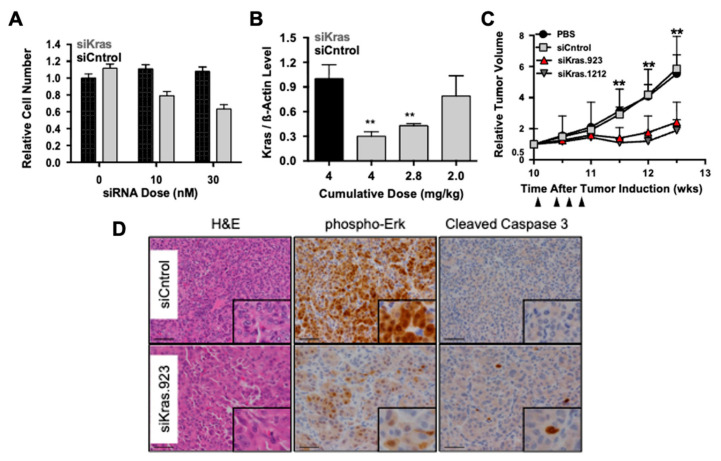
(**A**) KP cell number following incubation with nanoparticles containing siKras. Error bars are SD (n = 3 wells per group). (**B**) *Kras* mRNA expression in lung tumors isolated from KP mice following injection with nanoparticles containing siKras. **, *p* < 0.01. Error bars are SD (n = 4 mice per group, one tumor per mouse). (**C**) Relative lung tumor volume in KP mice, measured by microCT, following treatment with siKras nanoparticles. **, *p* < 0.01. Ten weeks after tumor initiation, mice were injected with 1.5 mg/kg of 7C1-siRNA every other day for four injections. Error bars are SD (n = 6 mice per group, n = 2 tumors per mouse). Arrowheads indicate time points of nanoparticle administration. (**D**) Representative histology and immunohistochemistry staining of lung tumors from mice treated with siKras nanoparticles (scale bar: 50 µm). Figure adapted from Xue et al. (*Proceedings of the National Academy of Sciences*, **2014**, *111*, E3553–E3561) [96] with permission.

Xue et al. investigated the delivery of KRAS-targeting siRNA using a polyethylenimine (PEI)-based nanoparticle system [96]. Their study utilized a KP lung cancer model, which represents lung adenocarcinoma driven by KRAS and p53 mutations. In cell culture experiments, treatment with the siRNA-loaded nanoparticles significantly reduced the number of KP tumor cells (Figure 5A). In a mouse model bearing KP tumors, systemic intravenous injection of the nanoparticle formulation resulted in a marked reduction in KRAS expression, indicating successful gene knockdown (Figure 5B). Compared with control siRNA, treatment with siKRAS nanoparticles reduced tumor volume over the course of 13 weeks following tumor induction (Figure 5C). Functional KRAS signaling, measured by phosphorylated ERK levels, was also reduced, while cleaved caspase-3 levels were significantly increased, indicating induction of apoptosis (Figure 5D). Yan et al. reported similar findings using a polyester-based nanoparticle platform encapsulating siRNA targeting the UBB gene [97]. Using the lung adenocarcinoma cell line HCC4017, the authors established a tumor xenograft mouse model and treated tumors with siUBB-loaded nanoparticles through direct tumoral injection. This treatment resulted in reduced tumor volume along with increased caspase-3 and caspase-7 activity, further demonstrating the potential of nanoparticle-mediated siRNA delivery for suppressing oncogenic pathways. Lv et al. explored a different approach using an MOF nanoparticle carrying siRNA targeting VEGF together with THPP and cisplatin, enabling combined gene therapy, photodynamic therapy (PDT), and chemotherapy [98]. This study highlights the modularity of nanoparticle platforms, which allows multiple therapeutic modalities to be integrated into a single delivery system. In an A549 lung adenocarcinoma mouse model, intravenous injection with MOF-siVEGF resulted in significant tumor reduction. Co-delivery of cisplatin with the MOF platform further enhanced tumor suppression, and the addition of PDT through light irradiation produced an even greater therapeutic effect.

These results demonstrate the potential of multifunctional nanoparticle systems that combine gene silencing with phototherapy and chemotherapy for enhanced treatment of lung cancer. Similar studies have also employed metal–organic framework platforms with tailored surface functionalization to improve RNA stability and therapeutic activity in lung cancer models [99].

### 4.2. miRNA

MicroRNAs (miRNAs) are small single-stranded RNA molecules that are endogenously expressed within cells and are processed into short double-stranded duplexes during maturation, similar to siRNA [100]. Unlike siRNA, which is typically introduced exogenously, miRNAs naturally function as regulators of gene expression. Once incorporated into the RISC protein, miRNAs bind loosely to complementary sequences on target messenger RNAs, leading to the repression of translation or degradation of the transcript. Because individual miRNAs can regulate multiple genes simultaneously, they play important roles in controlling cellular pathways involved in cancer development and progression. Peng et al. investigated the use of gold nanorods complexed with miR-320a-3p for targeting lung cancer [101]. The nanoparticles were functionalized with RGD peptides to enhance tumor targeting. In an A549 lung adenocarcinoma mouse model, intravenous administration of the miRNA-loaded nanorods resulted in a reduction in tumor volume compared with the scrambled RNA control. Gold nanomaterials also possess photothermal properties, allowing them to convert light into heat for cancer ablation [102]. When photothermal therapy was combined with the miRNA treatment, the authors observed an even greater reduction in tumor growth. Mechanistically, the therapeutic effect was associated with the suppression of the oncogenic transcription factor Sp1, a downstream target of miR-320a-3p, along with increased expression of the tumor suppressor PTEN and reduced levels of matrix metalloproteases. These findings illustrate the potential of inorganic nanoparticle systems to enable combination therapies by integrating gene therapy with photothermal treatment. Xue et al. further explored miRNA delivery using a PEI-based nanoparticle platform encapsulating miR-34a [96]. Following intravenous injection in a KP mouse model of lung adenocarcinoma, the nanoparticles produced a significant reduction in tumor volume. The therapeutic effect was associated with decreased tumor proliferation, as indicated by reduced expression of proliferation markers Ki67 and phosphorylated histone H3 (pHH3).

Together, these studies highlight the ability of nanoparticle systems to deliver miRNA therapeutics and modulate multiple oncogenic pathways involved in lung cancer progression.

### 4.3. mRNA

Messenger RNA (mRNA) therapy involves the delivery of mature RNA transcripts encoding a protein of interest, often one that is lost or inactivated during oncogenic transformation [103]. By restoring the expression of these proteins, mRNA therapeutics can counteract tumor development and progression. Compared with siRNA or miRNA, mRNA molecules are typically much larger, which presents additional challenges for intracellular delivery and stability. Le et al. demonstrated the use of a PBAE nanoparticle system encapsulating mRNA encoding bevacizumab, an antibody that binds vascular endothelial growth factor (VEGF) and inhibits angiogenesis [104]. Following intravenous injection in a non-small cell lung cancer mouse model bearing A549 tumors, the mRNA nanoparticle formulation produced greater tumor suppression than treatment with bevacizumab protein alone. At 27 days post-treatment, the nanoparticle-treated group exhibited reduced tumor volume, increased caspase-3 activity, indicating enhanced apoptosis, and fewer VEGF-positive cells, suggesting strong anti-angiogenic effects. Tang et al. developed a hybrid nanoparticle system designed to enhance cellular uptake and intracellular delivery of mRNA [105]. Their nanoparticle incorporated a cationic lipid component to promote endosomal escape, a hyaluronic acid shell to target CD44 receptors, which are highly expressed on many cancer cells, and mannose moieties to target mannose receptors that are also upregulated in certain tumor environments. The system encapsulated mRNA encoding the tumor suppressor protein p53. In experiments using the H1299 non-small cell lung cancer cell line, the nanoparticle formulation induced greater cytotoxicity than free p53 mRNA, accompanied by increased caspase-3 activation and apoptosis. These findings highlight the enhanced therapeutic effect of nanoparticle-mediated delivery compared with free mRNA, as the nanoparticle platform improves cellular uptake and promotes endosomal escape.

Together, these studies highlight the versatility of nanoparticle systems for mRNA delivery in lung cancer therapy. By enabling the expression of therapeutic proteins such as anti-angiogenic factors or tumor suppressors, nanoparticle-mediated mRNA delivery offers a modular strategy for targeting multiple oncogenic pathways.

### 4.4. CRISPR-Cas

Clustered Regularly Interspaced Short Palindromic Repeats (CRISPR) represents a gene-editing technology that utilizes Cas proteins, which function as endonucleases capable of cleaving either DNA or RNA, depending on the specific Cas variant [106]. In these systems, a guide RNA directs the Cas nuclease to a complementary target sequence, allowing for precise editing of the gene of interest. Because many cancers are driven by defined oncogenic mutations, CRISPR-based approaches have attracted attention as potential tools for directly correcting or disrupting disease-causing genes [107]. Several studies have explored nanoparticle-mediated delivery of CRISPR systems for lung cancer therapy. Marschhofer et al. demonstrated the application of a lipid nanoparticle (LNP) platform delivering Cas9 mRNA together with a single guide RNA targeting the KRAS G12S mutation in non-small cell lung cancer [108]. In their study, the LNP formulation was administered intratracheally to mice bearing A549 lung tumor models. The treatment produced measurable gene editing of the KRAS mutation, low in vivo toxicity, and significant disruption of KRAS-driven oncogenic signaling, as indicated by increased apoptotic staining on immunohistochemical analysis. Chen et al. reported similar findings using a PEI-based polymeric nanoparticle system carrying a CRISPR-Cas9 construct targeting the KRAS G12C mutation [109]. In vitro administration of this system resulted in the disruption of KRAS signaling and led to reduced proliferation and migration of A549 cells.

Together, these studies highlight the potential of nanoparticle-mediated delivery systems to enable CRISPR-based gene editing in lung cancer, providing a strategy for precise targeting of oncogenic mutations.

## 5. Conclusions

This review highlights the growing potential of RNA-loaded nanoparticle systems for pulmonary drug delivery. We first discussed the biological challenges associated with delivering RNA to the lungs, including fragmentation during aerosolization for inhalable therapies, the mucus and surfactant layers that limit particle penetration, and cellular barriers such as phagocytic uptake by alveolar macrophages, electrostatic repulsion at the cell membrane, and immune activation.

To overcome these obstacles, several nanoparticle-based delivery platforms have been developed. We reviewed representative systems, including lipid nanoparticles, polymeric nanoparticles, and inorganic nanomaterials, all of which can protect RNA cargo and improve its stability during delivery. These platforms can also enhance transport across mucus barriers, promote cellular uptake, and facilitate endosomal escape, helping to address many of the limitations associated with naked RNA therapeutics.

Unlike prior reviews that primarily summarize nanoparticle platforms and their applications, this work emphasizes how specific physicochemical and molecular design features govern interactions with pulmonary delivery barriers, linking nanoparticle chemistry directly to delivery performance.

Lipid nanoparticles remain the most clinically established platform, supported by their biocompatibility, efficient membrane interaction, and reliable endosomal escape, which together enable consistent RNA delivery in vivo. However, their reliance on self-assembly limits control over ligand presentation, making it difficult to precisely tune binding affinity or exploit multivalent avidity for targeted delivery. This lack of structural definition arises from the stochastic nature of self-assembly, which prevents precise control over nanoscale organization and limits the ability to systematically optimize structure-function relationships. Polymeric nanoparticles offer greater control over structure and functionalization through defined synthetic chemistry, enabling more deliberate tuning of targeting, stability, and barrier penetration. This flexibility comes with tradeoffs, including more complex synthesis, higher cost, and ongoing challenges related to biocompatibility. Inorganic nanoparticles provide structural stability and unique physical properties that can support multimodal applications, including imaging and externally triggered therapies. However, limited biodegradability, potential toxicity, and formulation challenges such as aggregation and low solubility restrict their broader clinical use.

Lipid nanoparticles currently represent the most promising platform for clinical application, supported by their demonstrated success in mRNA vaccines during the COVID-19 pandemic and their ability to achieve reliable RNA delivery at scale. While other platforms offer advantages in chemical control or functional versatility, LNPs have shown the most consistent balance between delivery efficiency, biocompatibility, and translational feasibility, making them the leading system for near-term therapeutic use. From a design perspective, further progress will depend on improving control over nanoparticle architecture, including more precise ligand presentation and multivalent organization, as well as developing more efficient and scalable synthetic strategies for complex delivery systems.

We also discussed recent therapeutic applications of RNA-loaded nanoparticles, using lung cancer as a key example. Multiple classes of RNA therapeutics, including siRNA, miRNA, mRNA, and CRISPR-Cas systems, have been explored to target oncogenic drivers and regulatory pathways involved in tumor progression. By encapsulating or complexing these RNA payloads, nanoparticle systems can enable more effective delivery and gene modulation within lung tissues. Taken together, these advances suggest that nanoparticle-mediated RNA delivery is a promising strategy for targeting lung pathologies. Continued progress in nanoparticle design and delivery methods may help enable more effective and less invasive therapies, including inhalable formulations capable of delivering RNA therapeutics directly to the respiratory tract. Continued advancement in this area will require aligning nanoparticle design with both the physicochemical and physiological constraints of the lung, including stability during aerosolization, transport across airway barriers, and efficient intracellular trafficking following uptake.

## Data Availability

No new data were created or analyzed in this study.
